# Metabolomic Evaluation of *Ralstonia solanacearum* Cold Shock Protein Peptide (csp22)-Induced Responses in *Solanum lycopersicum*

**DOI:** 10.3389/fpls.2021.803104

**Published:** 2022-01-07

**Authors:** Dylan R. Zeiss, Paul A. Steenkamp, Lizelle A. Piater, Ian A. Dubery

**Affiliations:** Department of Biochemistry, Research Centre for Plant Metabolomics, University of Johannesburg, Auckland Park, South Africa

**Keywords:** cold shock protein, defense, elicitor activity, hydroxycinnamic acids derivatives, metabolomics, phenylpropanoids and phenolics, plant immunity activation

## Abstract

*Ralstonia solanacearum*, the causal agent of bacterial wilt, is one of the most destructive bacterial plant pathogens. This is linked to its evolutionary adaptation to evade host surveillance during the infection process since many of the pathogen’s associated molecular patterns escape recognition. However, a 22-amino acid sequence of *R. solanacearum-*derived cold shock protein (csp22) was discovered to elicit an immune response in the Solanaceae. Using untargeted metabolomics, the effects of csp22-elicitation on the metabolome of *Solanum lycopersicum* leaves were investigated. Additionally, the study set out to discover trends that may suggest that csp22 inoculation bestows enhanced resistance on tomato against bacterial wilt. Results revealed the redirection of metabolism toward the phenylpropanoid pathway and sub-branches thereof. Compared to the host response with live bacteria, csp22 induced a subset of the discriminant metabolites, but also metabolites not induced in response to *R. solanacearum*. Here, a spectrum of hydroxycinnamic acids (especially ferulic acid), their conjugates and derivatives predominated as signatory biomarkers. From a metabolomics perspective, the results support claims that csp22 pre-treatment of tomato plants elicits increased resistance to *R. solanacearum* infection and contribute to knowledge on plant immune systems operation at an integrative level. The functional significance of these specialized compounds may thus support a heightened state of defense that can be applied to ward off attacking pathogens or toward priming of defense against future infections.

## Introduction

The immobile nature of plants exposes them to an environment filled with a diverse range of biotic—and abiotic stimuli and stressors. Since plants are the primary producers within most food systems, they are threatened by bacteria, fungi, viruses, insects, and herbivores. Plants synthesize a large variety of surface-located receptors that allow them to interact with the surrounding environment and defend themselves from unavoidable threats (Ranf, 2018; Malik et al., 2020). Plant innate immunity is thus reliant on cell-autonomous events, with these events displaying an overlapping similarity when compared to the immune system of animals. To compensate for the absence of an adaptive immune system, plants have evolved a greater pathogen recognition capacity (Freeman and Beattie, 2008; Dodds and Rathjen, 2010). The described pattern recognition receptors (PRRs) recognize and bind to molecules deemed foreign or non-self to the host (Sanabria et al., 2012), defined as microbial-associated molecular patterns (MAMPs). Exposure of the plant to a live pathogen would expose it to a consortium of different MAMPs, with different chemical signatures originating from peptide, carbohydrate and lipid-based structures (Boller and Felix, 2009; Sanabria et al., 2012). This results in the initiation of intracellular signaling cascades leading to a plant immune response in support of a broad-spectrum resistance that limits pathogen ingress and development (Malik et al., 2020). The perception of the MAMP molecules functions as an early warning system against pathogens and allows the rapid activation of the plant defense mechanisms.

The causal agent of the bacterial wilt disease, *Ralstonia solanacearum*, is regarded amongst the most destructive bacterial pathogens due to several behavioral, geographic and host factors (Mansfield et al., 2012). The disease has been difficult to contain due to the pathogen’s ability to evade host recognition. A contributing factor to the pathogen’s success would be that many of the prototypical MAMPs described in literature are not perceived by host plants (Wei et al., 2018)—allowing the pathogen to successfully infiltrate the host by bypassing many of the pre-existing defense mechanisms. For example, the polymorphic flagellin-derived flg22 sequence from *R. solanacearum* (flg22^Rso^), elicits no response in tomato (Wei et al., 2018, 2020), which sets back the development of strategies to limit the bacterial wilt disease.

However, the discovery of the cold shock protein (csp)-derived csp22 elicitor, reported to induce an immune response in several species of the Solanaceae, together with its corresponding PRRs, has opened new avenues of scientific exploration in the Solanaceae. The csps of bacteria are defined by intracellular hyperaccumulation in response to rapid temperature fluctuations of >10^°^C to assist with the challenges faced by the microorganism; which includes the rigidification of the cell membrane, inefficient protein folding, as well as a decreased efficiency in transcription and translation (Keto-Timonen et al., 2016; Wang et al., 2016). The highly conserved nucleic acid binding motif RNP-1 of csps (the 22-amino acid core or csp22) was found to function as MAMPs and to selectively induce immune responses in species belonging to the Solanaceae family (Zipfel, 2014; Wang et al., 2016). Initially, the phenomenon that Solanaceae plants possess an immunological detection method directed at the perception of membrane impermeable proteins naturally found in the cytoplasm of bacteria (as also applies to elongation factor Tu), came as a scientific mystery, and suggested the presence of a PRR for csp recognition located at the plant cell surface (Wang et al., 2016). The PRR of *Solanum lycopersicum* was identified by mapping the natural variation in csp22 perception between *S. lycopersicum* and *S. pennelli* and was named the cold shock protein receptor (CORE) (Wang et al., 2016; Wei et al., 2018). Similarly, in *Nicotiana benthamiana*, the PRR responsible for csp22 perception was named as the receptor-like protein required for csp22 responsiveness (NbCSPR). This PRR was found to associate with the brassinosteroid insensitive 1 (BRI1)-associated kinase upon elicitor treatment to confer bacterial resistance in an age-dependent and flagellin-induced fashion (Saur et al., 2016). These studies also underlined the biotechnological potential to augment immunity and defense by interspecies transfer of CORE/CSPR to other plant families (Wang et al., 2016).

This observation motivated for the investigation of downstream metabolic processes following csp22-elicitation in order to evaluate the functional significance of adaptive metabolome changes. In terms of “omics” levels, metabolomics is the final manifestation of integrated upstream biological information flow and thus the determinant of the eventual phenotype. Integration of data originating from metabolomics with transcriptome and/or proteome data offers gene-to-metabolite and protein-to-metabolite analysis in support of reliable understanding and interpretation of metabolism. Here, we monitored the effects of csp22-elicitation on *S. lycopersicum* using an untargeted metabolomics approach. Additionally, the study set out to discover trends that may indicate that csp22 treatment confers increased resistance to *R. solanacearum* in *S. lycopersicum.* Metabolomic analyses have shown potential in studies involving plant-pathogen interactions by revealing subtle metabolic alterations in response to biotic or abiotic perturbations and can subsequently be regarded as an adequate method of functionally investigating plant metabolism (Tugizimana et al., 2018; Zeiss et al., 2018, 2019; Castro-Moretti et al., 2020).

## Materials and Methods

### Plant Cultivation

Seeds from the tomato cultivar, “Star 9001” was obtained from a tomato breeding program for resistance against *R. solanacearum* (Stark Ayres, Pty. Ltd., Bredell, South Africa)^1^ and cultivated in germination mixture (Culterra, Muldersdrift, South Africa). Plants were grown under greenhouse conditions: a light/dark cycle of 12 h/12 h, with the light intensity set at 80 μmol/m^2^/s and the temperature regulated to between 22 and 24^°^C. Plants were rotated on a daily basis to prevent any positional effects.

### csp22 Peptide

The csp22 peptide elicitor with sequence ATGTVKWFNETKGFGFITPDGG (Wei et al., 2018), was synthesized at ≥90% purity (GL Biochem, Shanghai, China). A stock solution of the peptide elicitor was made to 1 mg/mL in 8 mM MgSO_4_ and used as a diluted sample during the inoculation procedures.

### DAB Histochemical Staining

The leaves of 6 w old mature tomato plants were treated with csp22 and subsequently stained with a 3,3′-diaminobenzidine (DAB, Sigma, St. Louis, United States) solution to visualize and detect the presence of hydrogen peroxide (H_2_O_2_). The protocol was performed with slight modifications as previously described (Bach-Pages and Preston, 2018). Briefly, the abaxial side of the leaves were treated with 500 nM csp22 by means of pressure infiltration and incubated for 30 min. The corresponding abaxial side of the leaves were treated with 8 mM MgSO_4_, which served as a negative control. Care was taken to avoid excess wounding or mechanical damage during pressure infiltration. The DAB solution (1 mg/mL in water, pH 3.8) was prepared 1 h before use. The leaves were excised from the plant and placed within a DAB solution under light at 23^°^C for 8 h with constant agitation. After the incubation period, the leaves were removed and immersed in boiling 70% ethanol for 10 min. After cooling, the leaves were transferred into absolute ethanol at room temperature and left overnight. The leaves were then imaged. The visible brown polymerized precipitate in the host tissue was produced as a result of the oxidation of DAB by H_2_O_2_.

### Oxidative Burst Luminescence Assay

Leaf disks (0.4 cm^2^) were punched out from fully expanded leaves using a cork borer. The leaf disks were floated adaxial side up on 200 μL MilliQ water in a white 96-well microtiter plate (Nunc, Roskilde, Denmark) which was placed under light at room temperature for 24 h. After the incubation period the water from each well was completely removed and replaced with a 100 μL of a master mix solution composed of: 34 μg/mL luminol (Sigma, St. Louis, United States) and 20 μg/mL horseradish peroxidase (Sigma, St. Louis, United States) and 1 μM csp22 in 8 mM MgSO_4_. Special consideration was taken to limit mechanical damage of the leaf disks during the floating disk and water removal steps. A negative control composed of the above-mentioned master mix, excluding the csp22 elicitor, was added. The negative control was supplemented with 8 mM MgSO_4_ to maintain sample volumes. Luminescence was measured every 2 min for 60 min using a Synergy HT Biotek microplate reader (Biotek Instruments, Vermont, United States). The luminescence data was exported to an excel file for further analysis. To account for natural variability three leaf disks per plant were taken. In total of 24 leaf disks were used per treatment condition.

### Plant Elicitation and Experimental Design

Six-week-old tomato plants were watered generously 5 h prior to elicitor inoculation to open leaf stomata and facilitate inoculation. Three plants, selected for uniform size and appearance, were reserved for each treatment/control condition prior to the treatment process. To ensure sample consistency, leaves from the fourth node branching point of the plants were selected for elicitor/control inoculation. The respective plants were treated with (500 nM) elicitor solution by pressure infiltration into the leaves using a blunt-ended syringe. Separate plants were treated with 8 mM MgSO_4_ functioning as a negative control. In each instance, the entire leaf surface was supplied with elicitor/control treatment solution to minimize biological variation. It should though be noted that the fragility and complex reticulate venation inherent with tomato leaves complicate the inoculation process and that care should be taken to avoid/limit wounding or mechanical damage. After inoculation, the plants were incubated for 16, 24, and 32 h, respectively. After each incubation time the inoculated leaves were harvested from the three selected plants, snap-frozen in liquid nitrogen to quench metabolic activity and stored at −80^°^C until further use. The experimental design consisted of three biological replicates (*n* = 3) that were created for each elicitor/control treatment at each time point (16, 24, and 32 h), thus constituting 18 biological sample groups (3 × 2 × 3) in total, covering all conditions.

### Metabolite Extraction

The leaf tissues frozen with liquid nitrogen were pulverized with a mortar and pestle. Two grams of leaf powder were extracted with 80% methanol in a 1:10 (w/v) ratio. The samples were sonicated twice in a sonicator bath for 30 min, with the temperature controlled at 20^°^C. Cell debris was pelleted with a bench top swinging-bucket centrifuge set at 5,525 × g and 5^°^C for 20 min. The supernatants were evaporated to 1 mL using a rotary evaporator at 55^°^C, carefully transferred into 2 mL microcentrifuge tubes and dried in a heating block overnight at 55^°^C. The samples were then reconstituted in 500 μL of 50% HPLC-grade Methanol: MilliQ water solvent (1:1, v/v). The 18 samples were filtered through 0.22 μm nylon syringe filters into vials fitted with 500 μL inserts and stored at 4^°^C until analyzed. Three pooled quality control (QC) samples consisting of aliquots of all samples, as well as 50% methanol blanks were included in the sample list. The QC samples were added to check sample stability, feature legitimacy, assess intensity drifts that occur during data the acquisition process, and monitor instrumental efficiency and robustness. Each of the 18 biological samples (that were prepared in triplicate as biological repeats) were analyzed in triplicate (technical repeats) on the UHPLC-MS instrument to gain precision and accuracy. Although the method described above has been widely used in scientific metabolomics literature, it should be noted that the extraction of highly polar compounds e.g., phosphates and sugars, highly non-polar compounds e.g., several lipid and sterol species, as well as volatile compounds may only partially extract or not at all. There are currently no extraction method that can recover the entire metabolome with a high level of robustness and reproducibility (Tugizimana et al., 2013). Previous literature has shown that the described method is able to recover many of the secondary metabolites of interest present within the tomato metabolome (Gómez-Romero et al., 2010; Roldan et al., 2014).

### Ultra-High Performance Liquid Chromatography Coupled to High Definition Mass Spectrometry

Two microliter of each sample extract was analyzed on an UHPLC-quadrupole time-of-flight (qTOF) high-definition MS system equipped with an electrospray ionization (ESI) source (Synapt G1, Waters Corporation, Manchester, United Kingdom). The analytes were separated on an Acquity HSS T3 reverse-phase column (2.1 × 150 mm × 1.7 μm; Waters Corporation, Milford, MA, United States) using a binary solvent system consisting of acetonitrile (Romil Chemistry, Cambridge, United Kingdom): MilliQ water, with both solvents containing 0.1% formic acid (FA, Sigma, Munich, Germany) and 2.5% isopropanol (Sigma, Munich, Germany). A gradient elution method was used over a 30 min run with a flow rate set to 0.40 mL/min. The elution was started at 2% (v/v) acetonitrile from 0 to 1 min, raised to 70% acetonitrile from 1 to 22 min, taken up to 95% from 22 to 23 min then kept constant at 95% acetonitrile from 23 to 26 min. The composition of the mobile phase was then reverted to 2% acetonitrile from 26 to 27 min, for column cleaning and equilibration from 27 to 30 min. The chromatographically separated metabolites were detected with the aid of a SYNAPT G1 high definition mass spectrometer (Waters Corporation, Manchester, United Kingdom) set to acquire data in both positive and negative ionization modes. The MS conditions were as follows: capillary voltage of 2.5 kV, sample cone voltage of 30 V, microchannel plate detector voltage of 1,600 V, desolvation temperature of 450^°^C, source temperature of 120°C, cone gas flow of 50 L/h, desolvation gas flow of 550 L/h, *m/z* range of 50–1,500, scan time of 0.2 s, interscan delay of 0.02 s, mode set as centroid. The lockmass flow rate was 0.1 mL/min, with leucine encephalin as reference calibrant (50 pg/mL, [M + H]+ = 556.2771 and [M – H]^–^ = 554.2615), continuously sampled every 15 s, producing an average intensity of 350 counts/scan in centroid mode, with typical mass accuracies between 3 and 5 mDa and a mass accuracy window of 0.5 Da. High purity Helium was used as desolvation-, cone-, and collision gas. The MS analyses performed in an unfragmented as well as four fragmenting experiments (MS^E^) simultaneously using ramping of the collision energy from 10 to 50 eV. The data acquisition at these collision energies was used to facilitate metabolite fragmentation and ease downstream structure elucidation and compound annotation. Each of the three biological replicates was analyzed in triplicate on the UHPLC-MS system, creating the technical replicates (*n* = 3). Thus, data for nine samples were obtained (*n* = 9) that was further processed by multivariate data analyses (MVDA).

### Metabolomics Data Processing and Analysis

The UHPLC-ESI-MS data sets were analyzed with Masslynx XS™ software (Waters Corporation, Manchester, United Kingdom), with the addition of other statistical programs for MVDA. The raw UHPLC-ESI-MS data was processed with MarkerLynx XS™ 4.2 software, with the following parameters: 0.60–21 min retention time (Rt) range of the chromatograms and *m/z* domain of mass range 50–1,500 Da. The Rts were allowed to differ by ±0.20 min and the *m/z* values by ±0.05 Da. The mass tolerance was 0.01 Da and the intensity threshold was 10 counts. Only the data matrices with noise level less than 50% (MarkerLynx cut off) were retained for downstream data analyses. The MarkerLynx application uses the patented ApexPeakTrack algorithm to perform accurate peak detection and alignment. Furthermore, MarkerLynx performs sample normalization, based on total ion intensities of each defined peak. Prior to calculating intensities, the software performs a modified Savitzky-Golay smoothing and integration (Zhou et al., 2012; Chen et al., 2013; Tugizimana et al., 2016).

The generated data matrices were imported into SIMCA (soft independent modeling of class analogy) software, version 14.0 software with the “omics” skin (Sartorius Stedim Data Analytics AB, Umeå, Sweden) for statistical analyses. To put all variables on equal footing, and adjust for measurement errors, the Pareto-scaling method was applied to data prior to chemometric modeling. A non-linear iterative partial least squares algorithm (built-in the SIMCA software) was used to handle missing values, with a correction factor of 3.0 and a default threshold of 50%. Unsupervised and supervised learning methods were applied. As part of the unsupervised methods, both principal component analysis (PCA) and hierarchical cluster analysis (HiCA) were applied. PCA is an unsupervised projection-based statistical method, used for reducing the multi-dimensionality of the data matrix. This is done by projecting the data matrix into lower dimensional space (2D) where global and qualitative visual representation of the observations can be observed. This results in the discovery and summarization of underlying clusters, trends, or sample outliers, as well as displaying the systematic variation present within the data matrix. Orthogonal projection to latent structures discriminant analysis (OPLS-DA modeling), as the chosen supervised method and binary classifier, was applied as the variable selection method to compare the samples of the elicitor treatment to that of the MgSO_4_ controls—leading to identification of metabolite features positively correlated to the discrimination between the two groups (Tugizimana et al., 2013). The OPLS-DA separates multivariate relationships into predictive (positively correlated to the csp22-treatment) and orthogonal (positively correlated to the control) variation. As a tuning procedure in computing the models, a sevenfold cross-validation (CV) method was applied (Tugizimana et al., 2016). Thorough model validation steps were consistently applied; and only statistically valid models were examined and used in data mining for metabolite annotation. For variable selection, the OPLS-DA loading S-plots were used. The loading plot displays an S-shape when the data is centered and Pareto-scaled. The loadings plot facilitates the identification of features with variability between groups (discriminating variables), i.e., variables situated at the upper right or lower left sections in the S-plot. Discriminant features with a |p(corr)| of ≥0.5 and a co-variance value of |(p1)| ≥0.05 were selected for further analysis (Trygg et al., 2007). It should be noted that these selection parameters are largely data dependent. These selection parameters were chosen based on the application of descriptive statistics (ANOVA) to the downstream metabolite features, where a statistical cut-off of *p*-value <0.05 was used. To avoid selection bias, the statistical significance of each potential marker was investigated with the application of univariate descriptive statistics. These analyses included the generation of average intensity values, standard deviations, *p*-values, fold changes, and each feature’s coefficient of variation in control and treatment samples. The overall selectivity of the OPLS-DA models was assessed by constructing receiver operating characteristic (ROC) curves. The ROC curves illustrated the supervised model’s ability to discriminate between features correlated to the different sample conditions. The predictive capacity of the supervised models was assessed with a *n* = 100 response permutation test. The permutation test consisted of comparing the *Q*^2^ obtained for the original data with the distribution of the *Q*^2^-values calculated during the randomly assigned permutations (Triba et al., 2014).

### Annotation of Metabolites

The chemical—and structural identities of the metabolites were elucidated using their respective mass spectral properties and patterns obtained during the MS analyses. MS spectral-based metabolite identification was performed based on sufficient and accurate mass fragment information, accurate calculation of each feature’s elemental composition and database searches for possible metabolite annotation. MassFragment, a built in Markerlynx software tool, was utilized for assigning possible structures to observed fragment ions of the precursor metabolite features using novel algorithms. The putative empirical formula of each statistically significant extracted ion peak (XIC) in the mass spectra was obtained and searched in databases (ChemSpider,^2^ Dictionary of Natural Products,^3^ PubChem,^4^ the KEGG Compound database,^5^ and Metacyc^6^ database for the identification of possible compound matches (Gómez-Romero et al., 2010; Mhlongo et al., 2016; Zeiss et al., 2018). The analysis and identification of lipids was facilitated using the Lipidmaps database.^7^ Metabolites were annotated and tentatively identified to level 2 of the Metabolomics Standards Initiative (MSI) (Sumner et al., 2007; Spicer et al., 2017).

## Results

The experiments were set up in such a way that the leaf tissues of the “Star 9001” tomato cultivar were pressure infiltrated with the *R. solanacearum-*derived csp22 peptide—the MAMP-perceived component of bacterial cold-shock proteins—and appropriately harvested at selected incubation time points, i.e., 16, 24, and 32 h post inoculation. The “Star 9001” cultivar was selected due to: (1) its apparent disease resistance toward *R. solanacearum* (Zeiss et al., 2018), as well as (2) minimal venation patterns present on the abaxial side of the leaves, which facilitated the pressure infiltration process. The research followed an untargeted metabolomics workflow to detect the elicitor-induced perturbations and subsequent metabolic patterns within the metabolome of *S. lycopersicum* that could be interpreted as a positive correlation toward a conferred resistant phenotype against *R. solanacearum*.

### Reactive Oxygen Species Production and Oxidative Burst

**FIGURE 1:**
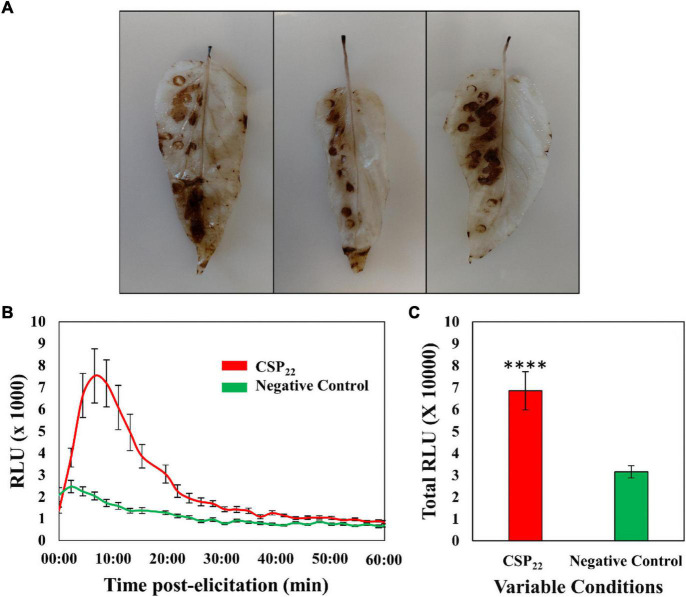
The generation of reactive oxygen species in the leaf tissue of *Solanum lycopersicum* cv. Star 9001 in response to csp22 (500 nM) elicitation. **(A)** The generation of H_2_O_2_ visualized using the peroxidase-dependent histochemical stain 3,3-diaminobenzidine (DAB) after 24 h incubation at room temperature. The left abaxial half of the leaves was infused with the csp22 elicitor while the right abaxial half served as the negative control for comparative purposes. **(B)** The total ROS production during the oxidative burst over 60 min as indicated as the integrated area under the curve, described as Σ relative luminescence units (RLU). The leaf disks were treated with a csp22 solution (red) and a negative 8 mM MgSO_4_ control (green). **(C)** The total kinetics of ROS production in 60 min after elicitor inoculation. Both experiments were replicated (DAB, *n* = 3 and luminescence *n* = 24), as three independent experiments. A pairwise Student’s *t*-test was performed to determine statistical significance. The asterisks indicate the degree of statistical significance (^****^p ≤ 0.0001).

The DAB staining protocol was performed, as a qualitative validation method, to verify that the elicitor was perceived by the plant and that a defense-related physiological response was triggered. The left abaxial side of the leaves was treated with the elicitor solution, while the right abaxial half was supplied with the MgSO_4_ control. The formation of a color product (Figure 1A) revealed the presence of H_2_O_2_ and associated ROS. The associated production of ROS *in planta* hints to the trigger of the oxidative burst and suggests the activation of initial plant immune responses. The time-dependent kinetics of the elicitor-linked oxidative burst was investigated using the chemiluminescence assay (Figures 1B,C). Univariate statistics were applied, in the form of a Student’s *t*-test, to determine the statistical significance (*P* ≤ 0.0001) between the two conditions. The data generated from the DAB stain and chemiluminescence assay confirmed the plant’s ability to perceive of the csp22 peptide elicitor. This is in contrast to the non-perception of the polymorphic flg22 from *R. solanacearum*, regarded as part of the pathogen’s immune evasion strategy (Wei et al., 2018, 2020).

### Chromatographic Separation and Mass Spectrometric Detection of Metabolites

**FIGURE 2:**
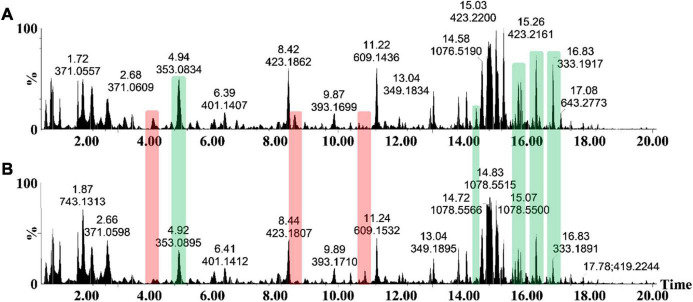
The UHPLC-MS analyses of methanol extracts from the csp22-treated (500 nM) *S. lycopersicum*, Star 9001 cultivar. A comparison of the metabolite profiles at the 16 h time interval—**(A)** treatment and **(B)** control—revealed concentration-related variances in peak intensities. The y-axis represents the relative abundance (%) of the metabolite fragments at their respective retention times (min). Changes in peak intensities (green) and/or the presence/absence of peaks (red) reflect differential variation in elicitor-induced leaf metabolism.

As an analytical platform, reverse-phase UHPLC-MS is especially suitable to reflect the overall phytochemical abundance of plants, including secondary/specialized metabolites which have a defensive or protective function (Tugizimana et al., 2013). Following leaf inoculation, harvesting and metabolite extraction with 80% methanol (able to extract a wide range of semi-polar and non-polar metabolites), the sample extracts were analyzed on such a high definition/accurate mass UHPLC-MS system. The base peak intensity (BPI) chromatograms highlighted the relative peak intensities and adequate resolution of individual peaks as well as the increased ionization of *S. lycopersicum* metabolites in ESI negative mode (Figure 2). Qualitative variation is reflected by the peak size—where the y-axis represents the relative intensity of metabolites on the x-axis at their respective Rts (min). Changes in peak intensities and/or the presence/absence of peaks reflect differential variation in csp22-induced leaf metabolism. A comparison of the chromatograms between the control and treated conditions highlights the induced metabolic perturbations over the described incubation points in the 20 min Rt window (Figures 2A,B).

### Data Analysis and Statistical Modeling

**FIGURE 3:**
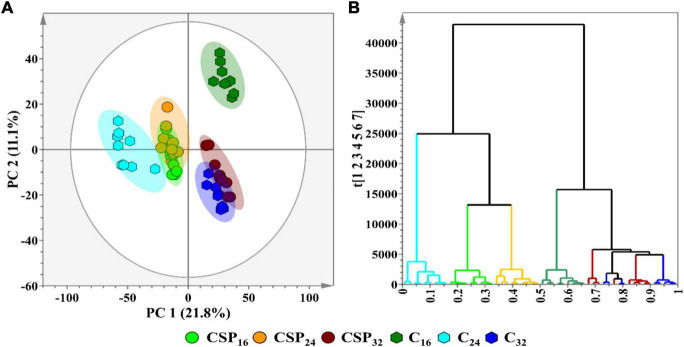
A principal component analysis (PCA) score plot revealing the group clusters within the data matrices of *S. lycopersicum* extracts from leaf tissue after csp22 elicitor treatment at the incubation time points (16, 24, and 32 h). **(A)** A 2D PCA scores plot illustrating the grouping of the observations. The PCA model had a calculated R_2_X (cum) value of 61.0% and a Q_2_ (cum) value of 50.3%. The ellipse on the score plot represents Hotelling’s T2 with a 95% confidence interval. **(B)** A Ward-linkage hierarchical cluster analysis (HiCA) dendrogram corresponding to **(A)**, showing the hierarchical outline of the data before and after treatment.

Multivariate data analysis was performed as the chosen method of data exploration by: (1) revealing trends within the metabolome across the incubation time points, and (2) detecting similarities/differences in the metabolite profiles of the treatment conditions. Processing of the chromatographic data yielded a data matrix containing the relative intensities of detected metabolite features (variables) present in each of the samples (observations). The data matrix was subjected to PCA (Figure 3A—an unsupervised projection-based statistical method, used for reducing the multi-dimensionality of the data matrix. By projecting the data embedded in the matrix into lower dimensions (2D), a global and qualitative visual representation of the observations can be observed—leading to the discovery and summarization of underlying clusters, trends, or sample outliers, thus displaying the systematic variation present within the data matrix. Additionally, the data matrices were subjected to HiCA (Figure 3B), an unsupervised hierarchical-based statistical method that was used complimentary to PCA. HiCA was performed: (1) to build a hierarchy of the sample observations and, (2) to subsequently reveal trends within the matrix that may be overlooked during PCA analysis. The HiCA model was constructed based on Ward’s linkage method, considering distance clusters between- and within-samples. The PCA scores plot (Figure 3A) revealed the partial overlap between some of the observational groups e.g., the 32 h control and treated groups. The partial overlapping pattern observation could be attributed to a single immune-inducing elicitor treatment rather than a cocktail of elicitors frequently associated with live pathogen infection. The immune response produced by csp22-treatment may leave the flux of some metabolic pathways unaffected resulting in metabolites with similar cellular levels in both the control and treated groups. This pattern of similarity in some metabolite classes may contribute to the partial overlapping pattern observed in the PCA scores plot.

**FIGURE 4:**
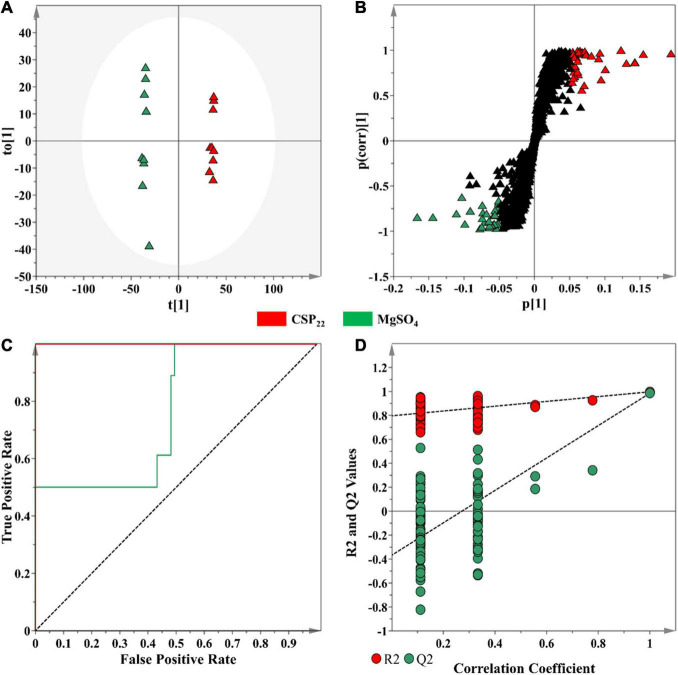
An OPLS-DA model for data processing of methanol extracts from tomato leaf tissue at the 16 h incubation time. **(A)** An OPLS-DA scores plot showing the group separation of control vs. treated (CSP_22_—Green vs. MgSO_4_–Blue) conditions. The calculated model yielded R^2^X (cum) = 46.1%, R^2^Y (cum) = 99.7% and Q^2^ (cum) = 98.5%. Model validation by 7-fold CV-ANOVA displayed a level of statistical significance with *p*-value = 8.502 × 10^–12^. **(B)** The corresponding OPLS-DA loading S-plot. Relevant variables on the loadings S-plot (at the extremes, x, y ≥0.05, 0.5), were selected and represent potential discriminating variables. **(C)** A receiver operating characteristic (ROC) curve summarizing the selective ability of a binary classifier (S-plot), with a classifier having a perfect discrimination producing a ROC curve that passes through the top left corner to indicate 100% sensitivity and specificity. **(D)** The response permutation test plot (*n* = 100) for the OPLS-DA model.

In contrast to unsupervised learning methods that evaluate the patterns within data, supervised methods are designed for the prediction, classification and discovery of biomarkers (Ren et al., 2015). OPLS-DA models were constructed, as the selected supervised statistical method, to inform on class separation (Figure 4A). The statistical significance for the observed class separation in the OPLS-DA models were measured by calculating the cross-validated analysis of variance (CV-ANOVA) *p*-values, as a tuning method, applying a cut-off of *p* <0.05 (Trygg et al., 2007; Eriksson et al., 2008). The *p*-values of each computed supervised model were tabulated (Supplementary Table 1). The corresponding OPLS-DA loadings S-plot (Figure 4B) showed variables that were positively correlated to class separation, i.e., csp22 treatment at the selected incubation time points. These multidimensional analyses were then applied toward the identification of significant features to consider for annotation.

Discriminant features with a | p(corr)| of ≥ 0.5 and a co-variance value of |(p1)| ≥0.05 were selected for further analysis (Trygg et al., 2007). These selection parameters were chosen based on the application of descriptive statistics (ANOVA) to the downstream metabolite features (Figure 4B), where a statistical cut-off of *p*-value <0.05 was used. This improved the selection of statistically relevant features, while simultaneously excluding the selection of false positives. The overall performance of the OPLS-DA models, in terms of selectivity, was assessed with the construction of a ROC curve (Figure 4C), showing that the supervised models, as binary classifiers, had perfect discrimination with regards to sensitivity and specificity (Supplementary Table 1). The predictive capacity of the supervised models was assessed with a *n* = 100 response permutation test (Figure 4D; Eriksson et al., 2008). The permutation test revealed that the presented supervised models had higher calculated *R*^2^ and *Q*^2^-values (Supplementary Table 1) in comparison to the model permutations, concluding that the OPLS-DA model obtained was statistically superior to the generated permutations (Eriksson et al., 2008; Saccenti et al., 2014). The corresponding sets of figures for the 24 and 32 h time points are presented as Supplementary Figures 1, 2.

### Selection of Discriminant Features Based on Multivariate Statistical Analysis Data

The OPLS-DA S-plot (Figure 4B) was used for the selection of metabolite features positively correlated to the elicitor treatment. The cut-off values of |p(corr)| of ≥ 0.5 and a co-variance value of |(p1)| ≥0.05 were determined based on the application of univariate descriptive statistics (the calculation of control and treatment averaged peak intensities, the standard deviation, the coefficient of variation, fold change between the two groups, and the *p*-value) on the selected features. Descriptive statistics were used to provide basic information and summarize the characteristics of the variables between the samples from control and treated tissues. Metabolite features that were found to contain a *p*-value >0.05 and a coefficient of variation >30 were removed from the generated list of features. Using this process, the optimal values threshold values for |p(corr)| and |(p1)| were determined.

### Metabolite Investigation

From 1,575 features (a combination of individual mass signals, including parental ions, isotopologs and possible MS fragments) initially acquired through ultra-high performance liquid chromatography coupled the mass spectrometry analyses, a total of 36 metabolites that were positively correlated to the csp22 treatment were putatively identified in the leaf tissue of *S. lycopersicum* (Table 1). The metabolites have been previously described in literature and databases related to tomato or plant species within the Solanaceae family. The metabolites were tabulated according to increasing Rt with corresponding *m/z* values. Among the identified metabolites, several of the phytochemicals belong to the phenylpropanoid class (including cinnamates, benzoates, flavonoids and coumarins), conjugates such as amides of hydroxycinnamic acids (HCAs) and chlorogenic acids (CGAs), organic acids and amino acid derivatives and lipid classes. The metabolites were annotated based on: (1) accurate mass, utilized for calculating an empirical formula, (2) analysis of each compound’s mass fragmentation, and (3) comparative analysis with existing literature.

**TABLE 1:** Annotation of metabolite signatures from the leaf tissue of *S. lycopersicum* displaying a positive correlation to the csp22 elicitor treatment at selected time intervals (16, 24, and 32 h).

#	Retention time (min)	Ionization	*m/z*	Putative identification	Chemical formula	Mass error (mDa)	Mass error (ppm)	Mass fragmentation	Metabolite class
1	0.94	[M–H]-	191.019	Citric acid	C_6_H_8_O_7_	–0.2	–1.0	173, 111	Organic acid
2	1.15	[M–H]-	191.019	Isocitric acid	C_6_H_8_O_7_	–0.2	–1.0	173, 111	Organic acid
3	1.17	[M+H]^+^	182.081	Tyrosine	C_9_H_11_NO_3_	–0.4	–2.2	165, 121	Amino acid
4	3.03	[M+H]^+^	188.069	Indole acrylate	C_11_H_9_NO_2_	–1.3	–6.9	142	Indole
5	3.22	[M–H]-	285.058	Gentisate pentoside	C_12_H_14_O_8_	–0.2	–0.7	153	Benzoic acid
6	3.45	[M–H]-	397.161	Benzoyl ornithine hexoside	C_18_H_26_N_2_O_8_	0.1	0.3	293, 235	Organic acid
7	4.12	[M–H]-	658.154	GSH-CGA	C_26_H_32_N_3_O_15_S	–1.0	–0.9	515, 466, 385, 191	Glutathione conjugate
8	4.70	[M–H]-	431.153	Benzyl alcohol dihexoside	C_19_H_28_O_11_	0.3	–0.9	269, 107	Benzoic acid
9	4.91	[M–H]-	367.158	Dihydroxy-dimethoxy prenylchalcone	C_22_H_24_O_5_	1.0	3.8	337, 299, 235	Flavonoid
10	4.94	[M–H]-	353.084	Caffeoyl quinic acid	C_16_H_18_O_9_	0.3	–7.9	191, 179, 135	CGA
11	4.99	[M+H]^+^	217.098	Cyclo methyltryptophan	C_12_H_12_N_2_O_2_	0.1	7.5	143	Indole
12	6.00	[M–H]-	293.121	Eugenyl cinnamate	C_19_H_18_O_3_	0.8	2.7	147	HCA
13	6.28	[M–H]-	355.101	Feruloyl hexoside	C_16_H_20_O_9_	–0.9	–2.5	193	HCA
14	6.79	[M–H]-	385.110	Feruloyl glucaric acid	C_16_H_18_O_11_	–1.1	–2.9	223	HCA
15	6.97	[M–H]-	385.075	Sinapoyl hexoside	C_17_H_22_O_10_	–0.6	–1.6	209, 193	HCA
16	7.31	[M–H]-	387.164	Hydroxyjasmonic acid hexoside	C_18_H_28_O_9_	–0.2	–0.5	225	Phytohormone derivative
17	8.62	[M–H]-	367.100	Feruloyl quinic acid	C_17_H_20_O_9_	–1.9	–5.2	191, 161	CGA
18	8.66	[M+H]^+^	177.054	Methylumbelliferone	C_10_H_8_O_3_	0.1	0.6	161, 106	Coumarin
19	9.86	[M–H]-	393.165	Acetyl tryptophan deoxyhexoside	C_19_H_26_N_2_O_7_	–0.6	–1.5	245, 203	Amino acid derivative
20	10.91	[M+H]^+^	695.365	N^1^, N^5^, N^14^-Tris-(dihydro-caffeoyl) spermine	C_37_H_49_N_4_O_9_	0.4	0.6	531, 474, 457, 293, 222	HCAA
21	10.99	[M–H]-	444.165	Coumaroyl tyramine hexoside	C_23_H_27_NO_8_	0.2	0.5	282	HCAA
22	11.22	[M–H]-	609.148	Rutin	C_27_H_30_O_16_	2.0	3.3	463, 301	Flavonoid
23	11.75	[M–H]-	490.170	Feruloyl dopamine hexoside	C_24_H_29_NO_10_	0.5	1.0	328	HCAA
24	12.97	[M–H]-	328.118	Feruloyl dopamine	C_18_H_18_NO_5_	0.3	0.9	177	HCAA
25	13.73	[M–H]-	282.112	Coumaroyl tyramine	C_17_H_17_NO_3_	0.9	3.2	147	HCAA
26	13.98	[M+H]^+^	792.599	Phosphatidyl choline (16:0/22:6)	C_46_H_82_NO_7_P	2.2	2.8	704, 625	Lipid
27	14.4	[M–H]-	453.23	Phosphatidyl glycerol (14:1)	C_20_H_38_O_9_P	2.0	4.4	379, 371, 299	Lipid
28	15.26	[M–H]-	423.221	Phosphatidic acid (8:0/8:0)	C_19_H_37_O_8_P	5.2	12.3	343, 297, 169, 89	Lipid
29	15.26	[M–H]-	459.198	Cryptochlorophaeic acid	C_25_H_32_O_8_	–4.5	–9.8	429, 415, 237	Benzoic acid derivative
30	15.33	[M–H]-	495.253	Hydroxycysteinylglycinyl eicosatetraenoic acid	C_25_H_40_N_2_O_6_S	1.9	3.7	451, 437, 351, 177	Lipid/amino acid derivative
31	15.59	[M–H]-	495.255	Hydroxycysteinylglycinyl eicosatetraenoic acid	C_25_H_40_N_2_O_6_S	2.1	4.2	451, 437, 351, 177	Lipid/amino acid derivative
32	15.92	[M+H]^+^	537.252	Phosphatidyl glycerol (18:1)	C_24_H_43_O_11_P	8.7	16.2	-	Lipid
33	17.40	[M–H]-	327.214	Trihydroxy octadecadienoic acid	C_18_H_32_O_5_	–1.0	–3.5	309	Lipid

*CGA, chlorogenic acid; HCA, hydroxycinnamic acid; HCAA, hydroxycinnamic acid amide.*

*The metabolites were annotated in both ionization modes as indicated using liquid chromatography coupled to mass spectrometry (UHPLC–MS). The metabolite features were annotated according to level 2 of the Metabolomics Standards Initiative (Sumner et al., 2007).*

**FIGURE 5:**
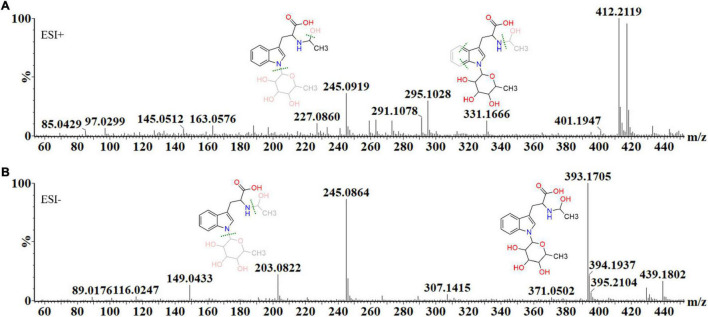
The mass spectral fragmentation pattern of acetyl tryptophan deoxyhexoside (C_19_H_26_N_2_O_7_) in **(A)** positive and **(B)** negative ionization modes. The MassFragment XS™ software, in conjunction with MS*^E^*, facilitated structural elucidation and compound identification, using the mass fragments in both ionization modes. In ESI (+) mode, the molecular ion is present as an adduct, 412.21 [M+H+NH_4_]^+^, while the main fragment ions are 145.05 [M+H-C_13_H_18_N_2_O_3_]^+^, 163.06 [M+H+C_10_H_18_NO_5_]^+^, 227.09 [M+H+C_6_H_16_O_5_]^+^, 295.10 [M+H-C_14_H_17_NO_6_]^+^ and 331.17 [M+H-CH_4_O_3_]^+^. In ESI (–) mode, the precursor ion is 393.17 [M–H]^–^, while the main fragment ions are 149.04 [M–H-C_11_H_18_NO_5_]^–^, 203.08 [M–H-C_8_H_14_O_5_]^–^ and 245.09 [M–H-C_6_H_12_O_4_]^–^, 307.14 [M–H-C_7_H_2_]^–^ and 439.18 [M–H+formic acid]^–^.

Below, the structural elucidation of acetyl tryptophan rhamnoside (a deoxyhexoside) is shown, demonstrating the steps involved in metabolite annotation. Each metabolite’s mass fragmentation pattern was investigated in both ionization modes, in conjunction with MS fragmentation at different energies (MS^E^), to illustrate how the MassFragment plugin of the MassLynx software facilitated the annotation of the fragment ions and verify the overall mass fingerprints. The elemental compositions of fragments were also calculated as a secondary method of validating each compound’s structural identity (Figure 5).

### Semi-Quantitative Analysis of Metabolites

**FIGURE 6:**
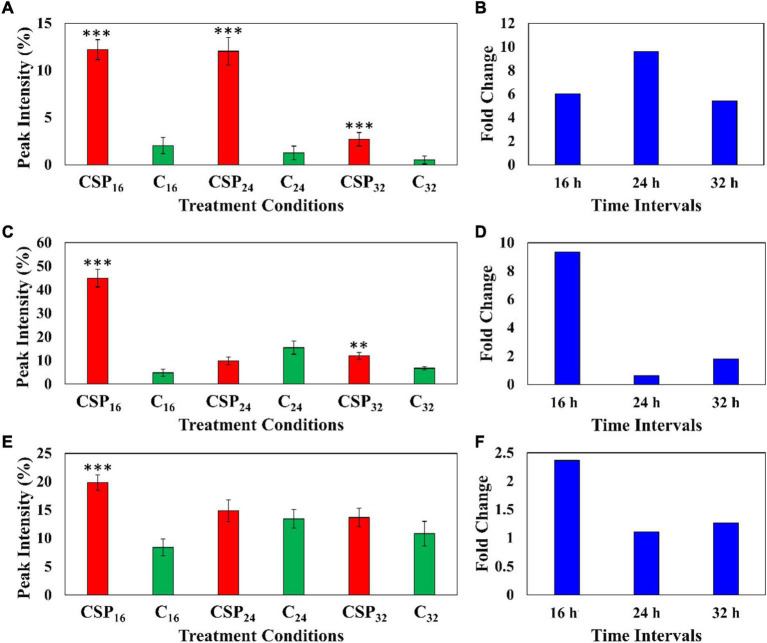
Fluctuating levels of ferulic acid derivatives in *S. lycopersicum* at selected incubation times (16, 24, and 32 h–red) after treatment with the csp22 peptide. The relative peak intensities of **(A)** feruloyl dopamine, **(C)** feruloyl quinic acid, **(E)** feruloyl hexoside in the treated samples (red). The corresponding fold changes **(B,D,F)** are displayed (blue) at the described time intervals. A MgSO_4_ control (C–green) was included for each time point as a comparative measure. Each data bar is presented as a mean value ($\bar{x}$of *n* = 9 samples) with the error bars indicating the calculated standard deviation (σ). A two-condition paired Student’s *t*-test was performed to compare the treatments with the MgSO_4_ control where the asterisks indicate levels of statistical significance (^**^p ≤ 0.001, ^***^p ≤ 0.0001).

The relative peak intensities of ferulic acid derivatives (feruloyl dopamine, feruloyl quinic acid and feruloyl hexoside) were compared between the MgSO_4_ control and csp22 elicitor treatments over the described incubation times (Figure 6). The data revealed that feruloyl dopamine biosynthesis (Figures 6A,B) was increased by a factor of ≥ 4 throughout the 16, 24, and 32 h incubation intervals, with the overall production of the compounds peaking at the 24 h time point. This observed result overlaps with literature relating the tomato-*Pseudomonas syringae* pathosystem (Zacarés et al., 2007). Feruloyl quinic acid (Figures 6C,D) exhibited its highest cellular abundance (a fold increase of ≥ 8) during the early 16 h time point which slowly decreased to a new cellular homeostasis level during the 24 and 32 h intervals. A similar trend was observed with the feruloyl hexoside derivative (Figures 6E,F). The hexoside conjugate showed the highest cellular abundance in the leaf tissue at the 16 h time point with a fold increase of ≥ 2. Following the 16 h time point, the levels of feruloyl hexoside decreased, returning to a newly established cellular homeostasis. The increased production of the feruloyl derivatives over the incubation period points to an elicitor-induced reprogramming at that occurs at the metabolome level. The observed trend indicates that the tomato leaf tissue produced most of its defense-associated compounds during the 16 h time point that decrease, either by metabolite degradation or conversion, during the 24 and 32 h incubation points.

**FIGURE 7:**
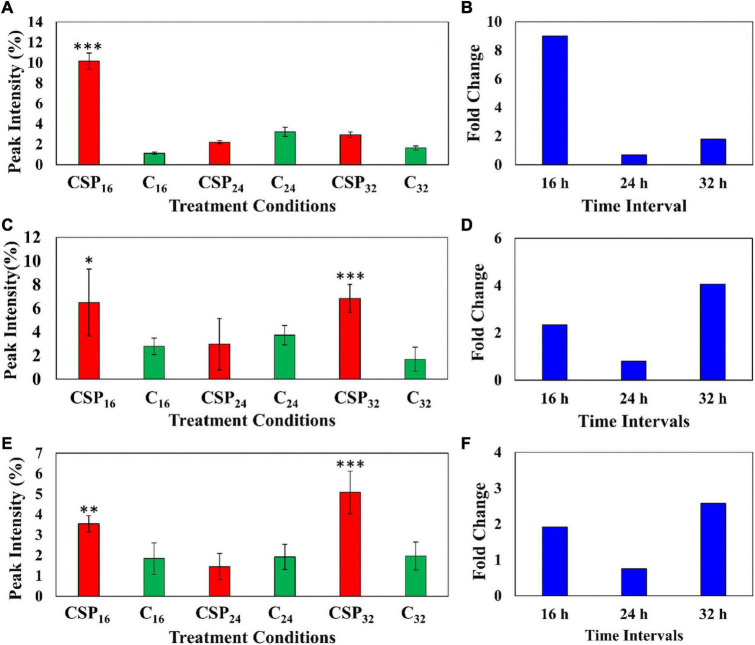
Fluctuating levels of selected metabolites in *S. lycopersicum* at selected incubation times (16, 24, and 32 h–red) after treatment with the csp22 peptide. The relative peak intensities of **(A)** methylumbelliferone, **(C)** hydroxy jasmonic acid hexoside, **(E)** coumaroyl tyramine hexoside in the treated samples (red). The corresponding fold changes **(B,D**,**F)** are displayed (blue) at the described time intervals. A MgSO_4_ control (C–green) was included for each time point as a comparative measure. Each data bar is presented as a mean value ($\bar{x}$ of *n* = 9 samples) with the error bars indicating the calculated standard deviation (σ). A two-condition paired Student’s *t*-test was performed to compare the treatments with the MgSO_4_ control where the asterisks indicate levels of statistical significance (* = *p* ≤ 0.01, ^**^ = *p* ≤ 0.001, ^***^ = *p* ≤ 0.0001).

The relative abundance of the coumarin, methylumbelliferone was compared in the elicitor treatment and the MgSO_4_ control over the three incubation times (Figure 7A). The data indicates that methylumbelliferone production was increased up until the 16 h interval followed by a decline to a new cellular homeostasis. The metabolite’s synthesis increased by a factor of ≥ 8 during the 16 h incubation interval indicating an early involvement in the host immune response (Figure 7B).

Similarly, the cellular levels of the phytohormone derivative, hydroxyjasmonic acid hexoside, were also monitored between the two conditions over the incubation intervals (Figures 7C,D). The phytochemical displayed two cellular increases during the 16 h and 32 h intervals, with a drop in the overall abundance during the 24 h point (Figure 7C). It should be noted that the metabolite levels at the 16 h time points, although statistically validated, demonstrated high levels of intra-sample variability. The jasmonic acid derivative showed a ≥ 2-fold change during the 16 h interval followed by a delayed but more intense ≥ 4-fold change in the 32 h interval (Figure 7D). A similar pattern was observed in the cellular levels of coumaroyl tyramine hexoside (Figures 7E,F). This HCA amide (HCAA) produced two statistically significant increases during the 16 and 32 h time intervals, while the 24 h point showed levels overlapping with the MgSO_4_ control. The compounds produced a ≥ 2-fold change during the 32 h time interval (Figure 7E).

## Discussion

Immune surveillance by the host involves an integrated network operating at different levels: MAMP perception and receptor activation, ROS production, calcium influx, activation of mitogen-activated protein kinase cascades, activation of G proteins, activation of transcription factors, etc. The intensity and/or sustainability of the oxidative burst can be determining factors that affect down-stream signal transduction events and recent studies have shown that pathogens manipulate host redox signaling to shape the eventual plant-pathogen interaction (Bleau and Spoel, 2021).

The metabolome of a biological system provides a functional readout of the cellular state, thus serving as direct signatures of up-stream biochemical events that define the dynamic equilibrium of metabolism and the correlated phenotype. The possibility of applying progressively improved metabolomic tools and approaches in plant-microbe studies has opened new ways to investigate the intricate details of how plant metabolism is activated and re-directed upon immune activation (Mareya et al., 2019; Tugizimana et al., 2019). Previously we have utilized metabolomics for comparative metabolic phenotyping of tomato plants for the identification of metabolic signatures associated with different response capacities conferred by phenotypic plasticity in cultivars differing in resistance to *R. solanacearum* (Zeiss et al., 2018). Metabolomic profiling of the tomato host response following infection by *R. solanacearum* revealed that the phenylpropanoid pathway, represented by flavonoids and HCAs, acts as the central hub of induced defenses (Zeiss et al., 2019). Increased concentrations and variability of metabolites associated with defense pointed to cultivar-specific variation in the speed and manner of resource redistribution between the host tissues. Differential metabolic signatures were linked to the resistant vs. susceptible metabolic phenotypes, underlying the defense metabolism and defining their defensive capabilities to *R. solanacearum* (Zeiss et al., 2019).

**FIGURE 8:**
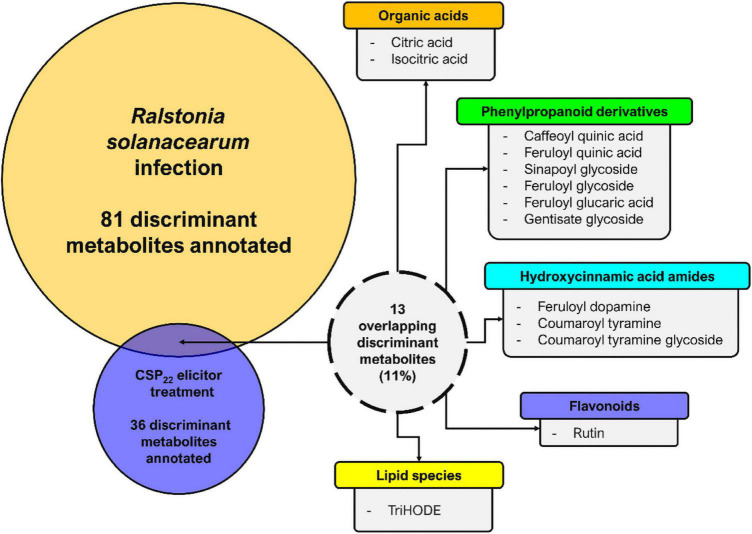
Metabolome perturbations and adaptive defense responses. A Venn diagram highlighting the partial overlap (%) of metabolites positively correlated to *R. solanacearum* and csp22 treatments in the leaf tissue of *S. lycopersicum*. The numerical values represent the annotated metabolites that are unique to each treatment and conversely are also shared. It should be noted that the experimental conditions—including the biological, technical, instrumental, and laboratory performance aspects—for both publications were identical and could thus be compared (Zeiss et al., 2019, 2021).

In this study, the changes to the composition of the tomato leaf metabolome again reflects an inducible resistant phenotype in the host. A comparative summary of discriminant metabolites (that could be positively annotated), present in leaf extracts from tomato plants infected with *R. solanacearum* vs. those present in leaf extracts from csp22-treated plants, are presented in Figure 8. It is evident that the single elicitor was able to trigger the synthesis of only a subset of metabolites compared to that of the consortium of MAMPs that are present during infection with live bacteria. Interestingly, csp22 was also able to elicit metabolites that were not annotated in the data set corresponding to the *R. solanacearum* infection (Zeiss et al., 2019).

In general, plants seem to mobilize similar chemical defense responses as reflected by activation of similar pathways leading to secondary metabolite synthesis. At a metabolite level, this might be reflected in enhanced synthesis of secondary metabolites with antimicrobial and antioxidant activities. Moreover, plants execute the triggered defense based on the perceived stimulus and the existing biochemical background operative in the naïve vs. stress-related conditions. Allowing for the dynamic nature of plant metabolism, qualitative and quantitative differences of specific metabolites or classes of metabolites within the broader metabolomic profiles, may modulate the eventual outcome of a host response to attempted infection (Mhlongo et al., 2021). These molecules (Table 1 and discussed below) were found to accumulate in varying amounts in the csp22-elicited leaves and exhibit differential accumulation patterns. These patterns indicate differential reprogramming over time (either high or low accumulation at specific time points, reflecting early-, late or oscillatory responses). The time-dependent reprogramming is an indication that plants re-adjust their metabolomes toward defense responses in order to ward off infection (Mhlongo et al., 2021). In the absence of a real infection by *R. solanacearum*, the levels of the csp22-induced metabolites decrease again with the establishment of a new cellular homeostasis.

**FIGURE 9:**
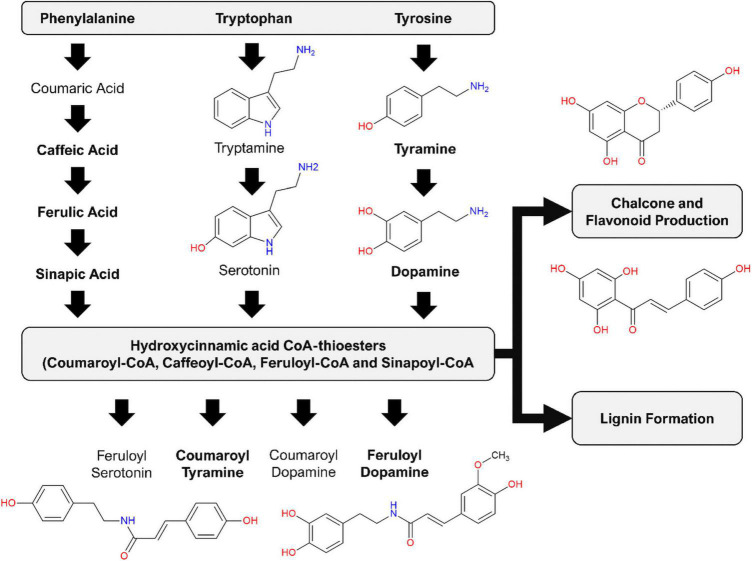
Induced production of hydroxycinnamic acid amides (HCAAs) in the leaf tissue of *S. lycopersicum* in response to csp-22 elicitation. Biosynthesis starts with increased levels of the aromatic amino acids: flux from chorismate to prephenate (for phenylalanine and tyrosine) and anthranilate (for tryptophan). The synthesis of the HCAAs involves the amide conjugation of the aromatic amino acids to hydroxycinnamic acid CoA-thioesters. The HCA thioesters can also feed into downstream pathways leading to the production of flavonoid derivatives as well as the formation of lignin. Compound names in bold were found in the present study, while the remaining phytochemicals have been reported in other studies related to the Solanaceae.

HCAs and—amides (HCAAs) (reviewed in Zeiss et al., 2021) are frequently associated with the metabolic stress responses of Solanaceous plants. In a comparative study of the effect of the MAMPs (lipopolysaccharides, chitosan, and flagellin-22) on *Nicotiana tabacum* cells, overlapping metabolomes were found that indicate common aspects where the phenylpropanoid pathway (modulated by both salicylic acid and the methyl ester of jasmonic acid) is activated by these elicitors and where HCA derivatives are consequently synthesized (Mhlongo et al., 2016). The results generated from this untargeted metabolomics study revealed that csp22-elicitation lead to the production of several ferulic acid derivatives. This is believed to be the first report of csp22-induced accumulation of HCAAs in tomato. The increased production of feruloyl dopamine at the described incubation periods highlights a significance in the tomato metabolome. Several research publications have described the production of HCAAs, such as coumaroyl- and feruloyl tyramine, coumaroyl- and feruloyl dopamine, coumaroyl- and feruloyl noradrenaline (Figure 9), implicated as phytoalexins of tomato after infection with bacterial pathogens (Von Roepenack-Lahaye et al., 2003; Zacarés et al., 2007; López-Gresa et al., 2011; Zeiss et al., 2019). The exact biological function of these compounds has not yet been fully elucidated (Zeiss et al., 2021). It has, however, been shown that these small molecules possess strong antioxidant activity, with radical scavenging abilities comparable if not better than that of the synthetic antioxidant butylhydroxytoluene (López-Gresa et al., 2011). The HCAA feruloyl dopamine has been shown to have good antimicrobial activity against *Pseudomonas syringae* (Zacarés et al., 2007). The quantity of scientific research surrounding these compounds remains limited. Transgenic studies involving *S. tuberosum* using Arabidopsis transporter genes have revealed that the rapid secretion of HCAAs positively correlates to a decreased ability of *Phytophthora infestans* spore germination (Dobritzsch et al., 2016).

Although caffeic acid and sinapic acid derivatives of HCAs were also amongst the identified variables, it is the HCA derivatives that carry a ferulic acid (*o*-methoxyphenol) substitution pattern that predominate: feruloyl hexoside, feruloyl glucaric acid, feruloyl quinic acid, feruloyl dopamine hexoside, feruloyl dopamine and eugenyl cinnamate. At an interactome level, from the pathogen’s perspective, the production of ferulic acid and other HCA derivatives represents an obstacle that must be overcome to facilitate the early stages of infection and host colonization. HCA degradation is a conserved trait that shields *R. solanacearum* from the chemical defenses of the hosts to promote pathogen virulence (Lowe et al., 2015). The study demonstrated that mutants lacking the enzymes required for HCA degradation were less virulent on tomato plants and simultaneously more susceptible to cellular toxicity (Lowe et al., 2015). It should be noted that this trait is specifically directed toward the degradation of HCAs, rather than the HCAAs. In addition, literature has documented that umbelliferone may serve as a plant-derived inhibitor capable of attenuating the Type 3 secretion system (T3SS) of *R. solanacearum* in tobacco, suppressing transcription of T3SS regulators and effectors (Yang et al., 2017). The *R. solanacearum* virulence factors are tightly regulated by a complex interlocking network involving quorum sensing and plant signals (Schell, 2000; Mole et al., 2007). It has been shown that the rapid and timely expression of these genes is a crucial and conserved virulence strategy employed by the bacterial wilt pathogen (Jacobs et al., 2012). The hydroxycoumarin umbelliferone has been shown to inhibit the growth and development of *Ralstonia* strains on solid media in a concentration-based manner (Yang et al., 2018). The same study also demonstrated that tobacco root irrigation with hydroxycoumarins 24 h prior to pathogen challenge delayed symptoms typically associated with bacterial wilt (Yang et al., 2018). With tobacco and tomato being phylogenetic relatives, both belonging to the Solanaceae, it is feasible to suggest that tomato also utilizes umbelliferone, in conjunction with other phytochemicals, to act as protective agents in the management of *R. solanacearum.*

As discussed above, the changes to the composition of the tomato leaf metabolome speaks to the resistant phenotype of the host. Jasmonic acid (JA) and its associated derivatives have been reported to be produced during biotic perturbations as key regulators of plant defense leading to several alterations in metabolic pathways resulting in the biosynthesis of secondary metabolites (Okada et al., 2015; Mhlongo et al., 2016). JA-mediated signaling pathways are linked to host resistance, prompting plant defense responses to external damage and pathogen infection, inducing gene expression typically observed in a resistant phenotype (Ruan et al., 2019). Hydroxylation and glycosylation are common steps used by plants to reduce or attenuate the bioactivity of particular metabolites *in vivo*. The finding of a hydroxyjasmonic acid derivative in the leaf extracts in response to csp22 elicitation thus indicates prior JA activity (Miersch et al., 2008; Hamany Djande et al., 2020) in the intracellular signaling cascade that leads to the production of defense-related compounds (e.g., the HCAAs). This can contribute to the host plant displaying an accelerated and heightened state of plant defense. Using an untargeted approach, this upregulation of the JA- and coumaroyl tyramine derivatives could be observed over the 16, 24, and 32 h incubation intervals, bolstering the knowledge described in literature (Mhlongo et al., 2016). Based on the results obtained, it would be feasible to suggest that JA production induced by csp22 leads to a more resistant host phenotype that may be more equipped against future infection against a broad-spectrum of pathogens including *R. solanacearum*.

## Conclusion

The results generated from this untargeted metabolomics study revealed that csp22 perception by leaf tissue of the tomato plant results in metabolome perturbation and the redirection of cellular metabolism to lead to the production of defensive and protective metabolites. This can be regarded as part of a functional metabolic strategy to cope with external environmental threats such as combating biotic stressors. These biochemical perturbations, primarily involving the phenylpropanoid pathway and sub-branches thereof, are linked to a csp22-triggered oxidative burst and associated downstream signal transduction events. The metabolites identified as signatory biomarkers include HCAs, HCAAs, and ferulic acid derivatives, emphasizing the role of the phenylpropanoid pathway as the central hub of induced defenses in tomato. Previous studies have associated the production of HCAAs (with antimicrobial and antioxidative properties) with biotic stress and elicitor treatments, and a dedicated function for these phytochemicals during plant-pathogen interactions is starting to emerge. Furthermore, the csp22-induced production of the hydroxyjasmonic acid derivative bolsters evidence of the phytohormone’s involvement in intracellular signaling that leads to the production of defense-related compounds, contributing to an enhanced defensive capacity within the plant tissue. This heightened state may equip the host plant to better defend itself against present pathogen attack or future infection against a broad-spectrum of pathogens. These results, from a metabolomics perspective, thus support a role for csp22 treatment of tomato in order to increase resistance to *R. solanacearum* infection and contribute to greater insights into the mechanism of perception of *R. solanacearum*, aiding multi-omics approaches to generate resistance.

## Data Availability Statement

The datasets presented in this study can be found in online repositories. The names of the repository/repositories and accession number(s) can be found below: https://www.ebi.ac.uk/metabolights/MTBLS3708.

## Author Contributions

ID: conceptualization, funding acquisition, project administration, and supervision. DZ, PS, and ID: methodology and investigation. DZ: writing—original draft. ID and LP: writing—review and editing. All authors have read and agreed to the published version of the manuscript.

## Conflict of Interest

The authors declare that the research was conducted in the absence of any commercial or financial relationships that could be construed as a potential conflict of interest.

## Publisher’s Note

All claims expressed in this article are solely those of the authors and do not necessarily represent those of their affiliated organizations, or those of the publisher, the editors and the reviewers. Any product that may be evaluated in this article, or claim that may be made by its manufacturer, is not guaranteed or endorsed by the publisher.

## Footnotes

1
www.starkeayres.co.za

2
www.chemspider.com

3
https://dnp.chemnetbase.com/

4
http://pubchem.ncbi.nlm.nih.gov/

5
https://www.genome.jp/kegg/compound/

6
https://metacyc.org/

7
https://www.lipidmaps.org/
