# Supplementary material for: Metabolomic Evaluation of Ralstonia solanacearum Cold Shock Protein Peptide (csp22)-Induced Responses in Solanum lycopersicum
Source: Front Plant Sci. 2022 Jan 7;12:803104. doi: 10.3389/fpls.2021.803104 (PMC8780328; doi:10.3389/fpls.2021.803104)
Supplement: Supplementary file 1 [file Data_Sheet_1.docx]

Metabolomic Evaluation of *Ralstonia solanacearum* Cold Shock Protein Peptide (csp22)-Induced Responses in *Solanum lycopersicum*

***Dylan R. Zeiss, Paul A. Steenkamp, Lizelle A. Piater, Ian A. Dubery****

*Research Centre for Plant Metabolomics, Department of Biochemistry, University of Johannesburg, P.O. Box 524, Auckland Park 2006, South Africa.*

**SUPPLEMENTARY DATA**

**Table S1.** Statistical validation of the computed OPLS-DA models corresponding to the tomato leaf elicitor treatment data matrices. The calculated R^2^X(cum), R^2^Y(cum) and Q^2^(cum) values for each of the three OPLS-DA models are presented. The R^2^ and Q^2^ values of the permutation analysis (*n =* 100 random permutations) are compared and shown to be significantly lower than the original values. The *p-*value of a 7-fold CV-ANOVA was shown to indicate statistical significance of each investigated model. (MgSO = MgSO_4_ controls, Csp = Cold-shock peptide; all at time intervals of 16 h, 24 h and 32 h post elicitation).

| **Model** | **N** | **R^2^X(cum)** | **R^2^Y(cum)** | **Q^2^(cum)** | **Permutation** | | **ROC AOC** | | ***p*-value of CV-ANOVA** |
| --- | --- | --- | --- | --- | --- | --- | --- | --- | --- |
|  |  |  |  |  | **R2** | **Q2** | **Control** | **Elicitor** |  |
| ESI (Negative) Supervised Models | | | | | | | | | |
| MgSO_16_ *vs.* Csp_16_ | 18 | 46.1% | 99.7% | 98.5% | 0.78 | -0.36 | 76.3% | 100% | 8.502 x 10^-12^ |
| MgSO_24_ *vs*. Csp_24_ | 18 | 42.6% | 98.9% | 9.33% | 0.84 | -0.33 | 97.4% | 68.7% | 1.606 x 10^-7^ |
| MgSO_32_ *vs*. Csp_32_ | 18 | 26.3% | 99.6% | 92.0% | 0.94 | -0.39 | 78.9% | 78.6% | 5.108 x 10^-7^ |
| ESI (Positive) Supervised Models | | | | | | | | | |
| MgSO_16_ *vs.* Csp_16_ | 18 | 44.5% | 99.8% | 98.6% | 0.79 | -0.45 | 100% | 73.6% | 6.432 x 10^-12^ |
| MgSO_24_ *vs*. Csp_24_ | 18 | 66.1% | 99.9% | 97.7% | 0.92 | -0.38 | 67.2% | 38.2% | 2.223 x 10^-8^ |
| MgSO_32_ *vs*. Csp_32_ | 18 | 25.4% | 99.3% | 88.9% | 0.87 | -0.40 | 90.0% | 84.4% | 4.241 x 10^-6^ |

**
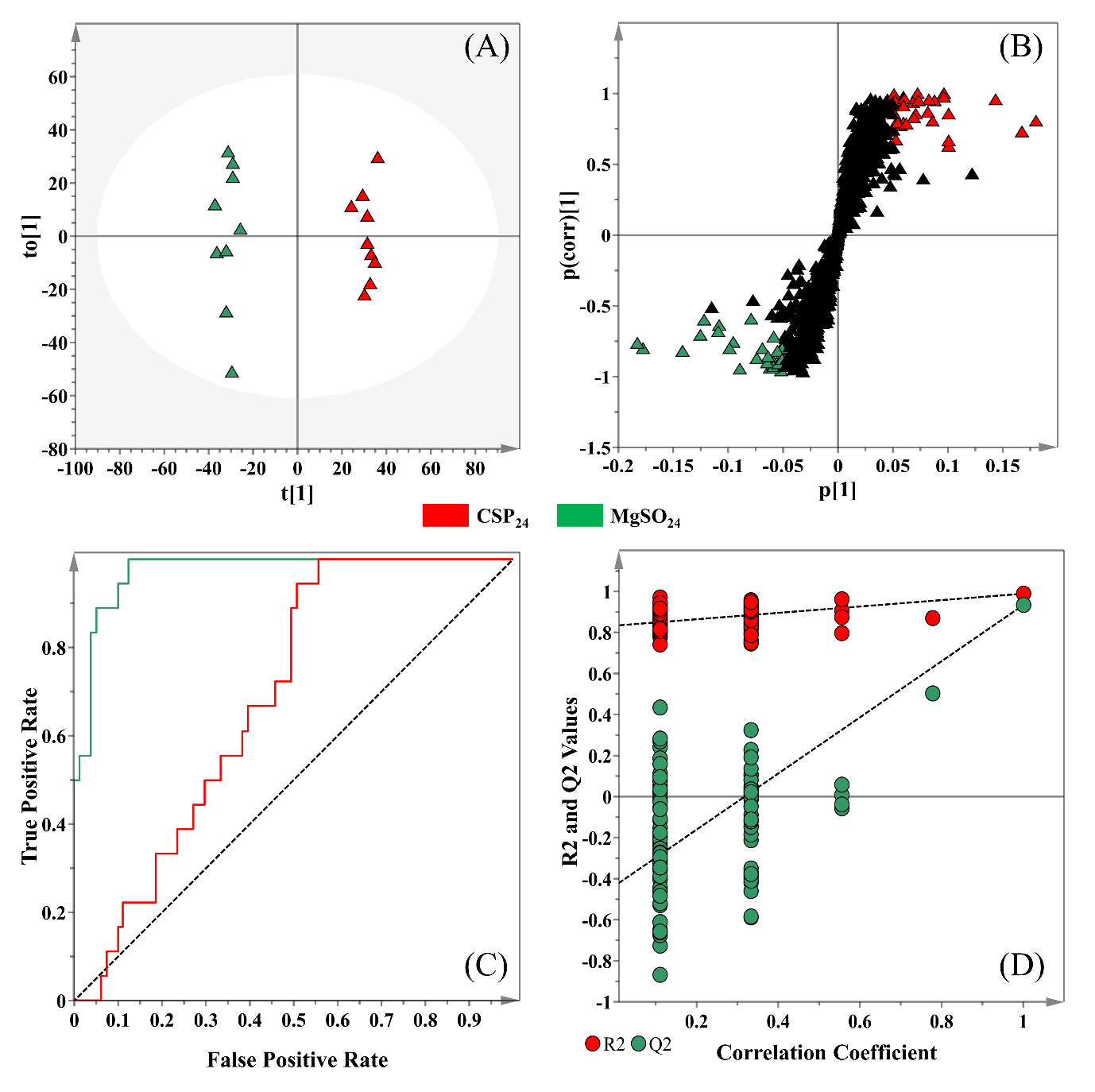
**

**Figure S1.** An OPLS-DA model for data processing of methanol extracts from tomato leaf tissue (MgSO_4_ control and Csp22 elicitor treatments) at the 24 h incubation time. **(A)** An OPLS-DA scores plot showing the group separation of control *versus* treated (Csp_24_ - Green *versus* MgSO_24_ - Blue) conditions. The calculated model yielded R^2^X (cum) = 46.1%, R^2^Y (cum) = 99.7% and Q^2^ (cum) = 98.5%. Model validation by 7-fold CV-ANOVA displayed a level of statistical significance with *p*-value *=* 1.606 x 10^-7^. **(B)** The corresponding OPLS-DA loading S-plot. Relevant variables far out in the loadings S-plot (x, y ≥ 0.05, 0.5) were selected and represent possible discriminating variables. **(C)** A receiver operating characteristic (ROC) curve summarizing the selective ability of a binary classifier (S-plot), with a classifier having a perfect discrimination producing a ROC curve that passes through the top left corner to indicate 100% sensitivity and specificity. **(D)** The response permutation test plot (*n* = 100) for the OPLS-DA model.

**
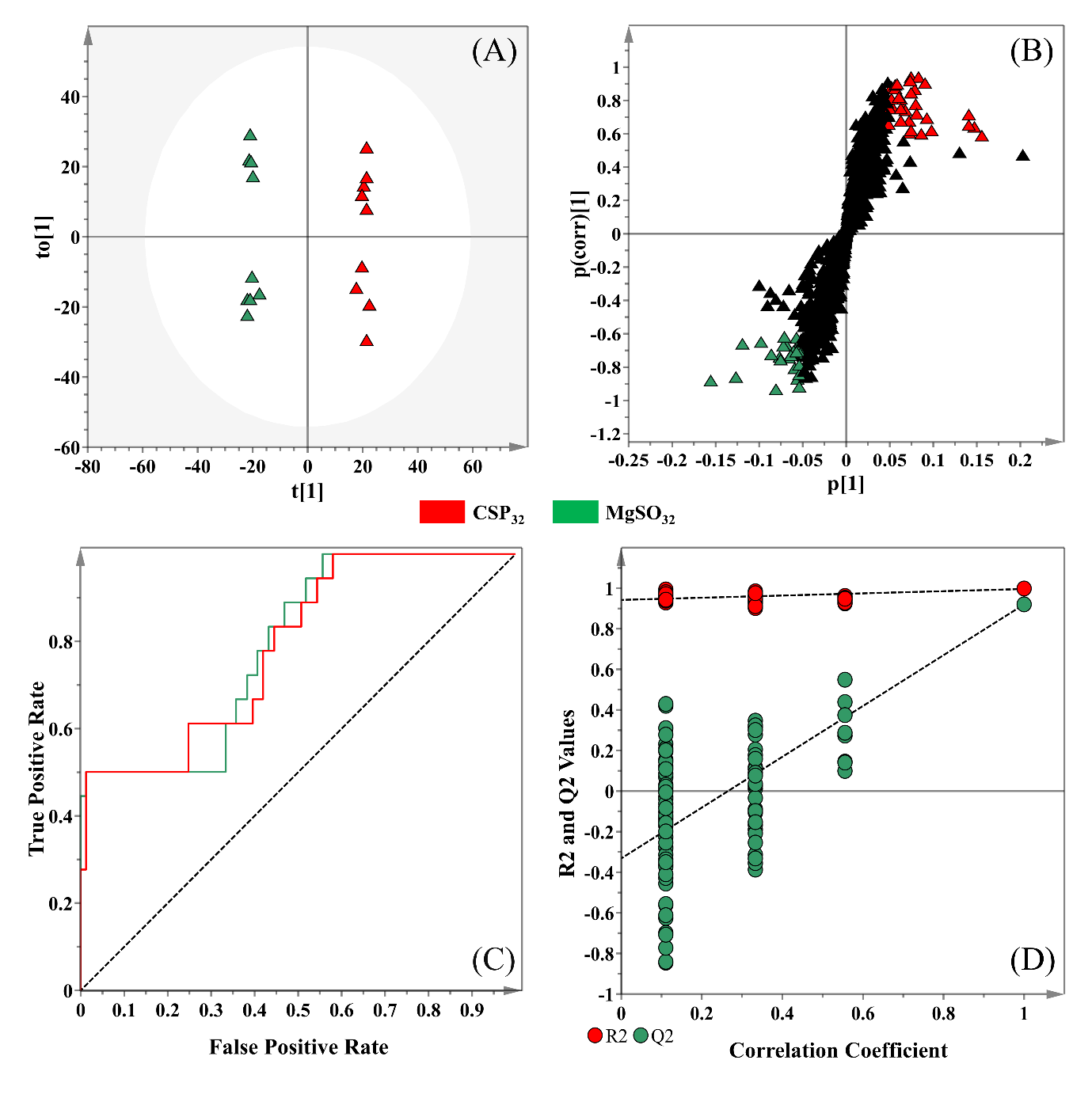
**

**Figure S2.** An OPLS-DA model for data processing of methanol extracts from tomato leaf tissue (MgSO_4_ control and Csp22 elicitor treatments) at the 32 h incubation time. **(A)** An OPLS-DA scores plot showing the group separation of control *versus* treated (Csp_32_ - Green *versus* MgSO_32_ - Blue) conditions. The calculated model yielded R^2^X (cum) = 46.1%, R^2^Y (cum) = 99.7% and Q^2^ (cum) = 98.5%. Model validation by 7-fold CV-ANOVA displayed a level of statistical significance with *p*-value *=* 5.108 x 10^-7^. **(B)** The corresponding OPLS-DA loading S-plot. Relevant variables far out in the loadings S-plot (x, y ≥ 0.05, 0.5) were selected and represent possible discriminating variables. **(C)** A receiver operating characteristic (ROC) curve summarizing the selective ability of a binary classifier (S-plot), with a classifier having a perfect discrimination producing a ROC curve that passes through the top left corner to indicate 100% sensitivity and specificity. **(D)** The response permutation test plot (*n* = 100) for the OPLS-DA model.
